# Advances in Osteoporosis Therapy: Focus on Osteoanabolic Agents, Secondary Fracture Prevention, and Perioperative Bone Health

**DOI:** 10.1007/s11914-023-00793-8

**Published:** 2023-06-08

**Authors:** Paul J. Kostenuik, Neil Binkley, Paul A. Anderson

**Affiliations:** 1 Phylon Pharma Services; School of Dentistry (Adjunct), University of Michigan, Newbury Park, CA USA; 2 Osteoporosis Clinical Research Program, University of Wisconsin, Madison, WI USA; 3 Department of Orthopedics and Rehabilitation, University of Wisconsin, Madison, WI USA

**Keywords:** Teriparatide, Abaloparatide, Romosozumab, Orthopedic surgery, Spine surgery

## Abstract

**Purpose of Review:**

This review summarizes recently published data and other developments around osteoanabolic osteoporosis therapies in patients with very high fracture risk, including those undergoing bone-related surgery.

**Recent Findings:**

Two osteoanabolic agents, abaloparatide and romosozumab, were recently approved for treatment of patients with osteoporosis at high fracture risk. These agents, along with teriparatide, are valuable for primary and secondary fracture prevention. Orthopedic surgeons are well positioned to facilitate secondary fracture prevention via referrals to fracture liaison services or other bone health specialist colleagues. This review aims to help surgeons understand how to identify patients with sufficiently high fracture risk to warrant consideration of osteoanabolic therapy. Recent evidence around the perioperative use and potential benefits of osteoanabolic agents in fracture healing and other orthopedic settings (e.g., spinal fusion and arthroplasty) in individuals with osteoporosis is also discussed.

**Summary:**

Osteoanabolic agents should be considered for patients with osteoporosis at very high fracture risk, including those with prior osteoporotic fractures and those with poor bone health who are undergoing bone-related surgery.

## Introduction

Osteoporosis is a systemic skeletal disease characterized by low bone mass and microarchitectural deterioration of bone tissue that leads to bone fragility and a consequent increase in fracture risk [1]. The WHO operationally defined osteoporosis based on bone mineral density (BMD) *T*-score [2], but most osteoporotic (i.e., low-trauma) fractures in older adults occur in those with non-osteoporotic BMD [3], and an osteoporotic fracture history is a stronger determinant of fracture risk than is low BMD [4••]. Fracture risk is particularly elevated after a recent fracture, which is one of several clinical factors indicating very high fracture risk (VHFxR). VHFxR has long been recognized as an indication for osteoporosis pharmacotherapy [5].

**Fig. 1:**
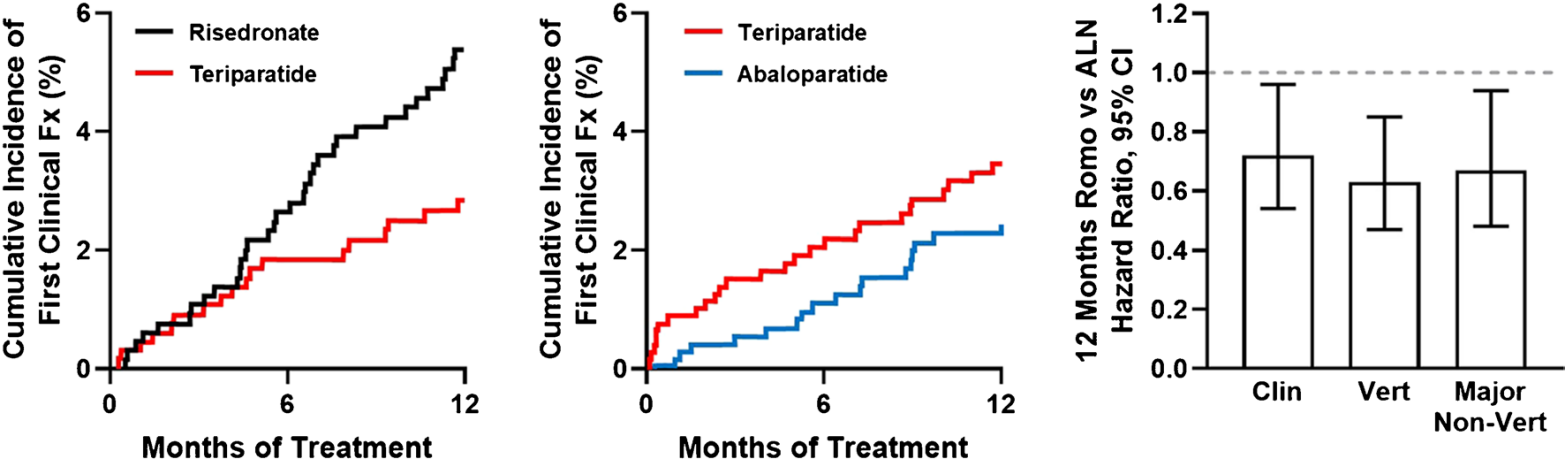
Evidence for early fracture risk reduction with osteoanabolic agents versus bisphosphonates. *Left* panel shows early separation of Kaplan–Meier curves for clinical fractures (Fx) in postmenopausal women with severe osteoporosis treated with teriparatide or risedronate during the first 12 months of the VERO trial (adapted from [7]). VERO showed that 24 months of teriparatide significantly reduced the risk of clinical, vertebral, and multiple vertebral fractures versus risedronate. *Middle* panel shows clinical fracture Kaplan–Meier curves for women with PMO treated with abaloparatide or open-label teriparatide over the first 12 months of the ACTIVE trial (adapted from [8]). In light of the VERO trial results (left panel), these data indirectly imply an early benefit of abaloparatide versus risedronate. ACTIVE showed that 18 months of abaloparatide significantly reduced the risk of major osteoporotic fractures versus teriparatide. *Right* panel shows hazard ratios and 95% confidence intervals for clinical, vertebral, and major non-vertebral fractures in women with PMO treated for 12 months with romosozumab versus alendronate in the ARCH trial, with *P* values of 0.027, 0.003, and 0.019, respectively (adapted from [10])

**Fig. 2:**
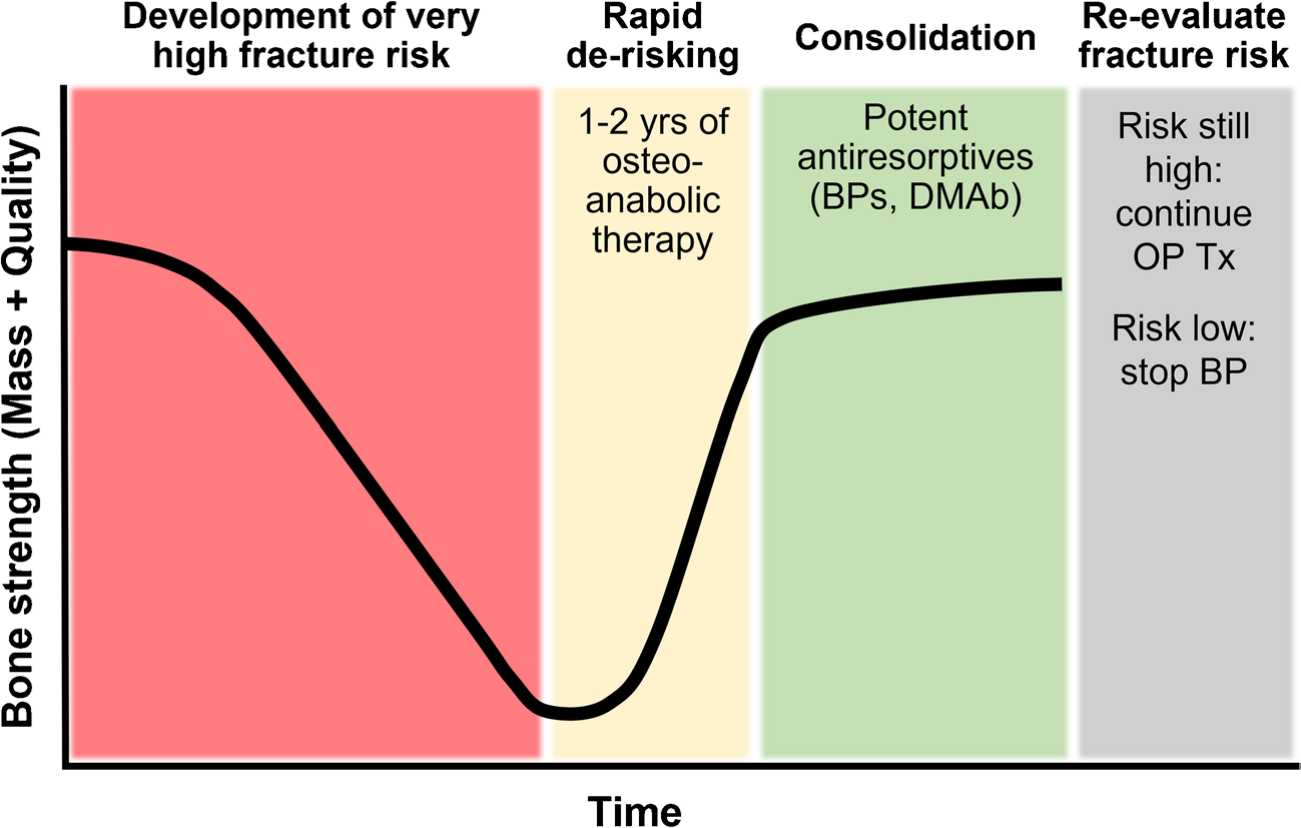
Potential approach for treating patients with osteoporosis at very high fracture risk (adapted from [51]). Many of these risk factors are listed in Table 1. Clinical practice guidelines recommend 1–2 years of osteoanabolic therapy (e.g., 12 months of romosozumab or 18–24 months of teriparatide or abaloparatide) to rapidly increase bone mass and reduce fracture risk. The resulting BMD gains should be consolidated (i.e., preserved or further increased) by follow-on therapy with potent antiresorptives such as amino-bisphosphonates (BPs, e.g., alendronate or zoledronic acid) or denosumab (DMAb). Fracture risk should be re-evaluated thereafter to determine the need for continued therapy; if fracture risk remains high, consider additional treatment with potent antiresorptives or re-treat with osteoanabolics. If fracture risk is sufficiently reduced, bisphosphonate therapy may be stopped or temporarily suspended, though discontinuation of denosumab without another follow-on therapy (e.g., bisphosphonate or romosozumab) leads to rapid bone loss and an increased risk of vertebral fractures

There is little debate regarding the net value of osteoporosis pharmacotherapy for secondary fracture prevention [6••], but treatment rates after fracture are low. Orthopedic surgeons are well positioned to play an important role in secondary fracture prevention, being the bone health specialists fracture patients are mostly likely to see and having high credibility in communicating the burden of fractures and the value of interventions. Pharmacotherapeutic options include antiresorptive agents (e.g., bisphosphonates or denosumab) that increase BMD by reducing bone resorption, osteoanabolic agents (teriparatide, abaloparatide) that increase BMD by stimulating osteogenesis, and a mixed agent (romosozumab) possessing both mechanisms, which will be referred to herein as an osteoanabolic agent. Osteoanabolics are increasingly used first line in patients with VHFxR (Fig. 1) because they are superior to antiresorptives for rapidly increasing BMD and reducing fracture risk [7–10] (Fig. 2), and because they produce greater BMD gains when used before rather than after antiresorptives [11•]. The potent antiresorptive agents denosumab (an anti-RANKL antibody) and zoledronic acid (ZOL, an intravenous bisphosphonate) are also recommended options for patients with VHFxR [4••].

Orthopedic surgeons are also increasingly focused on perioperative bone health optimization (BHO) in surgery candidates with osteoporosis, with osteoanabolics being valued for their potential to rapidly improve bone stock and promote bone regeneration [12]. This review summarizes recent advances in osteoanabolics, focusing on patients with prior fractures and those undergoing orthopedic surgery who have or are suspected of potentially having osteoporosis.

## Indications and Mechanisms of Action for Osteoanabolics

### PTH Receptor Agonists (Teriparatide, Abaloparatide)

PTH receptor (PTHR) agonists increase bone formation, BMD, and bone strength by activating PTH receptors on osteoblasts. Teriparatide, also known as recombinant PTH(1–34) and by the brand names FORTEO, FORSTEO, or Teribone, is a 34-amino-acid N-terminal fragment of full-length PTH(1–84). This self-administered daily injection is indicated to treat women with postmenopausal osteoporosis (PMO) at high risk for fracture, to increase bone mass in men with osteoporosis at high fracture risk, and to treat men and women with osteoporosis associated with glucocorticoid use at high fracture risk.

Abaloparatide (brand names TYMLOS or Eladynos) is a synthetic 34-amino-acid peptide with homology to PTH-related peptide (PTHrP). This self-administered daily injection is indicated to treat women with PMO at high fracture risk and men with osteoporosis at high fracture risk. Abaloparatide stimulates bone formation by activating PTH receptors, but with lesser increases in bone resorption compared with teriparatide, leading to greater increases in hip and femoral neck BMD and a lower risk of major osteoporotic fractures versus teriparatide [8].

BMD gains achieved with PTHR agonists are rapidly lost after their discontinuation unless patients initiate another osteoporosis therapy, typically a potent antiresorptive such as alendronate (ALN, an oral bisphosphonate), ZOL, or denosumab (Fig. 2). Potential side effects with PTHR agonists include hypercalcemia, nausea, and orthostatic hypotension.

### Sclerostin Inhibitors (Romosozumab)

Romosozumab (brand name EVENITY), the first marketed sclerostin inhibitor, is a humanized monoclonal antibody administered by health care professionals via two monthly s.c. injections for 12 months. Romosozumab is indicated to treat women with PMO who have high fracture risk or have failed or are intolerant to other osteoporosis therapies. Romosozumab is not approved to treat men with osteoporosis. Romosozumab binds and inhibits sclerostin, a soluble inhibitor of Wnt signaling that regulates systemic bone mass. Romosozumab dramatically but transiently stimulates bone formation and persistently reduces bone resorption, leading to greater hip and spine BMD gains versus potent antiresorptives [10, 13] or teriparatide [14]. One year of romosozumab followed by 1 year of ALN is superior to 2 years of ALN in reducing vertebral, clinical, non-vertebral, and hip fracture risk [10]. Similar to PTHR agonists, BMD gains are reversible after discontinuing romosozumab unless followed by potent antiresorptives (Fig. 2). Potential side effects with romosozumab include osteonecrosis of the jaw, atypical femoral fractures, and major adverse cardiovascular events (MACE, e.g., myocardial infarction or stroke) [15].

## Updates on Osteoanabolics in Patients with Osteoporosis

### Teriparatide

The phase 3 teriparatide trial in women with PMO [8] was not powered to evaluate hip fractures, but meta-analysis of 23 teriparatide studies in patients with osteoporosis showed a 56% relative reduction in hip fractures with teriparatide versus placebo or active comparators [16]. Analyses of 4 prospective observational studies showed lower hip fracture risk in patients receiving > 12 months versus < 6 months of teriparatide [17•].

Patient registry and postmarketing surveillance studies of teriparatide users indicate no increased risk of osteosarcoma [18, 19], which prompted changes to the US prescribing information for teriparatide: the boxed osteosarcoma warning was removed, and language on lifetime use of teriparatide beyond 2 years changed from “not recommended” to “should only be considered if a patient remains at or has returned to having a high risk for fracture” (https://pi.lilly.com/us/forteo-pi.pdf).

### Abaloparatide

The randomized placebo-controlled “ATOM” trial in men with osteoporosis showed that 12 months of abaloparatide increased lumbar spine, total hip, and femoral neck BMD versus placebo, with a safety profile consistent with previous trials [20]. These results supported the recent US FDA approval of abaloparatide for the treatment of men with osteoporosis at high risk of fracture.

Post hoc analyses from the phase 3 ACTIVE trial in women with PMO showed that more abaloparatide-treated subjects experienced clinically meaningful BMD gains at the hip, femoral neck, and lumbar spine versus placebo or teriparatide [21•]. Other post hoc ACTIVE data indicate greater increases in cortical volumetric BMD (vBMD) of the total hip, femoral neck, trochanter, and femoral diaphysis with abaloparatide versus placebo or teriparatide [22, 23•]. Recent cardiovascular safety analyses from ACTIVE indicated that abaloparatide was associated transiently increased heart rate, a small decrease in blood pressure, and no increased risk of cardiac adverse events, MACE, or heart failure [24].

ACTIVE included an open-label extension (ACTIVExtend) wherein the original abaloparatide and placebo groups transitioned to ALN. Post hoc analyses showed a lower vertebral fracture rate over 18 months of abaloparatide treatment during ACTIVE versus 24 months of ALN during ACTIVExtend among those previously receiving placebo [9], implying that abaloparatide reduces vertebral fractures versus ALN.

Iliac crest bone biopsies from women with PMO showed that 3 months of abaloparatide increases trabecular, endocortical, intracortical, and periosteal bone formation [25], findings that are newly reflected in the product label’s mechanism of action section (https://radiuspharm.com/wp-content/uploads/tymlos/tymlos-prescribing-information.pdf). Another abaloparatide label change is removal of the boxed warning regarding osteosarcoma risk. Other abaloparatide updates include its approval in Japan and the EU for the treatment of osteoporosis, and a new drug submission filing for marketing authorization in Canada.

An expert opinion on osteoanabolic therapy for women with PMO suggests that abaloparatide may be considered an ideal first option for patients at very high risk of vertebral fracture [26•].

### Romosozumab

Post hoc analyses from the placebo-controlled phase 3 FRAME trial in women with PMO show that romosozumab caused relatively greater reductions in osteoporotic fractures in subjects with higher baseline risk of major osteoporotic fractures (MOF) [27]. Iliac crest bone histomorphometry analyses from FRAME showed that romosozumab rapidly increases modeling-based bone formation, indicating stimulated osteogenesis on previously quiescent (non-resorbing) bone surfaces [28].

Supporting romosozumab use before rather than after antiresorptives, a retrospective observational study in Japan showed that spine BMD responses to romosozumab were blunted by > 1 prior years of denosumab or oral bisphosphonate therapy [29]. Moreover, phase 2 data show that romosozumab-induced BMD gains were preserved for 2 years by a single follow-on ZOL infusion [30]. Supporting re-treatment with romosozumab, the phase 2 data in postmenopausal women showed that a second 12-month course of romosozumab administered after 12 months of placebo induced BMD gains that were similar to those achieved during the first 12 months of de novo romosozumab therapy [31].

An expert opinion on osteoanabolic therapy for women with PMO suggests that romosozumab may be considered an ideal initial therapy for patients at very high risk of non-vertebral fractures, with abaloparatide a close second choice [26•].

## Updated Osteoporosis Guidelines, Taskforce/Consensus Reports, and Expert Opinions

Updates to several osteoporosis clinical practice guidelines (CPGs) highlight the importance of identifying patients with VHFxR who may warrant osteoanabolics. Prominent among these is an update issued jointly by AACE/ACE^1^ [4••], which is also highlighted in the AAOS 2021 guidelines on managing hip fractures in the elderly [32]. AACE/ACE 2020 CPGs communicate various risk factors for VHFxR, including certain fracture histories, with osteoanabolics receiving a grade A recommendation for initial use by patients with VHFxR. Updated Endocrine Society CPGs also endorse osteoanabolics for patients at VHFxR, including those with severe or multiple osteoporotic fractures [33]. Table 1 summarizes these and other contemporary criteria for the use of osteoanabolics, most of which are clinical features that are ascertainable during routine clinic visits.

**Table 1:** Criteria endorsed by professional societies and groups for the use of osteoanabolics in patients with osteoporosis. In many cases, denosumab and zoledronic acid are also endorsed

Criterion	Society or group endorsing criterion	Refs
Very high fracture risk (various definitions)	AACE/ACE, NAMS, NOF/OP-Canada/ANMM, ESCEO/IOF, NOGG	[4••, 34, 36, 37, 38•]
Prior fracture	NAMS	[34]
Recent fracture	AACE/ACE, NAMS	[4••, 34]
Multiple fractures	AACE/ACE	[4••]
History of severe osteoporotic fracture	ENDO	[33]
History of vertebral fracture	ASBMR	[6••]
Recent vertebral fracture	NOGG	[38•]
Multiple vertebral fractures	ENDO	[33]
Multiple vertebral fractures or hip fracture and spine or hip *T*-score ≤ − 2.5	BHOF	[35•]
History of hip fracture	ASBMR	[6••]
Recent hip fracture	NOGG	[38•]
Fracture while taking drugs that harm bone	AACE/ACE, NOGG	[4••, 38•]
Recent fracture while on glucocorticoids	NOGG	[38•]
Fracture while on approved OP therapy	AACE/ACE, NAMS	[4••, 34]
Bone loss while on antiresorptive therapy	NAMS	[34]
High risk of falls	AACE/ACE	[4••]
History of injurious falls	AACE/ACE	[4••]
Very high fracture probability by FRAX®	AACE/ACE	[4••]
BMD *T*-score below − 3.0	AACE/ACE, NAMS	[4••, 34]
BMD *T*-score below − 2.5 with fracture history	ENDO	[33]
Patient with OP undergoing spinal instrumentation	AANS/CNS	[39•]
Adult with OP undergoing elective spine surgery	Expert multidisciplinary consensus panel	[40•]

*OP*, osteoporosis; *NOF*, National Osteoporosis Foundation; *OP-Canada*, Osteoporosis Canada; *ANMM*, Academia Nacional de Medicina de Mexico; *AACE*, American Association for Clinical Endocrinology; *ACE*, American College of Endocrinology; *NAMS*, North American Menopause Society; *ESCEO*, European Society for Clinical and Economic Aspects of Osteoporosis, Osteoarthritis and Musculoskeletal Diseases; *IOF*, International Osteoporosis Foundation; *NOGG*, National (UK) Osteoporosis Guideline Group; *ASBMR*, American Society for Bone and Mineral Research; *ENDO*, Endocrine Society; *BHOF*, Bone Health and Osteoporosis Foundation; *AANS*, American Association of Neurological Surgeons; *CNS*, Congress of Neurological Surgeons

An ASBMR taskforce on secondary fracture prevention advises that osteoanabolics are appropriate initial therapies for individuals with vertebral fractures [6••]. The report advises that treatment of patients with hip or vertebral fractures should not be delayed for BMD testing, which partly reflects real-world challenges in timely access to DXA. We note, however, that DXA has value for monitoring treatment effects and promoting patient compliance, and DXA remains important in some regions (including the USA) for insurance coverage of osteoporosis pharmacotherapy.

Updated CPGs from NAMS advise that vertebral or hip fracture history is sufficient to diagnose osteoporosis irrespective of BMD or other risk factors, with osteoanabolics recommended as initial therapy in patients with VHFxR, including those with prior and especially recent fractures or fractures while on antiresorptives [34]. A recent clinician’s guide from BHOF (formerly NOF) on preventing and treating osteoporosis recommends osteoanabolic therapy for patients with VHFxR, defined as multiple spine fractures, or hip fracture with a *T*-score of − 2.5 or lower at lumbar spine or hip [35•]. An expert opinion report issued jointly by the NOF (USA), Osteoporosis Canada, and ANMM (Mexico) also recommends osteoanabolics in patients with VHFxR [36].

Updated ESCEO/IOF CPGs recommend osteoanabolics for patients at high risk of fracture and advise that women over 65 years old with an osteoporotic fracture history can be considered for treatment without BMD testing [37]. An update from NOGG (UK) recommends osteoanabolics for patients with VHFxR, including those with recent fractures, especially of the spine, hip, and humerus [38•].

AANS/CNS CPGs regarding osteoporosis therapy before spine surgery highlight relationships between osteoporosis and adverse postoperative events, and preoperative teriparatide was recommended for patients with osteoporosis undergoing spinal instrumentation [39•]. An expert consensus report on osteoporosis assessment and treatment in adults undergoing elective spine surgery concluded that bone health should be considered in all such patients, especially those at higher risk (e.g., > 65 years old or history of fracture) [40•]. Osteoanabolics were recommended as first-line therapy if not contraindicated, for at least 2 months when used preoperatively (up to 6 months preoperatively for elective multi-level procedures) and at least 8 months when used postoperatively [40•].

In summary, osteoanabolic therapy is recommended as first-line therapy for patients with VHFxR by multiple organizations and experts, with no evident controversy or dissent.

## Fractures Beget Fractures: Rationale for Secondary Fracture Prevention

Prior osteoporotic fractures increase future fracture risk independent of age and BMD. Fracture risk increases rapidly after certain fractures [41, 42] and may remain elevated as long as 10–25 years [43, 44], with the greatest risk during the first 1–2 years [41–43, 45–49, 50•]. Prior fractures are a major determinant of fracture risk by the online fracture risk calculator FRAX® (https://frax.shef.ac.uk/FRAX/), and a forthcoming FRAX update (FRAX_PLUS_) [51] may include fracture recency and prior fracture number as algorithm refinements [52•, 53•].

Treatment rates for secondary fracture prevention are troublingly low, even in patients hospitalized after severe osteoporotic fractures [45–48, 50•, 54]. Suboptimal secondary fracture prevention may partly relate to impressions among some surgeons that the follow-up and management of osteoporosis in post-fracture patients are the responsibility of primary care providers [48]. That viewpoint has near-term rationales [55] but potentially adverse long-term implications. For example, retrospective studies show that patients with recent osteoporotic fractures before total hip or knee arthroplasty (THA, TKA) have higher odds of periprosthetic fractures, prosthesis-related complications, and secondary osteoporotic fractures; yet, such patients rarely received osteoporosis pharmacotherapy [54, 56•]. Fortunately, surgeons are particularly effective at motivating post-fracture patients to initiate osteoporosis therapy [55, 57], with recent fractures serving as “teachable moments” [55]. Many post-fracture patients accept surgeon recommendations for osteoanabolics [58••], and one orthopedic group reported a 23% reduction in secondary fractures among patients with recent osteoporotic vertebral compression fractures (OVCF) who took osteoanabolics versus a 15% reduction among those receiving antiresorptives [59]. Such findings align with randomized trial data showing better fracture risk reduction with osteoanabolic agents (Fig. 1). The American Orthopedic Association (AOA) recognizes that orthopedic surgeons are well suited to assume greater responsibility for osteoporosis assessment and treatment [55, 60•], which may involve patient referrals to fracture liaison services (FLS) or bone health specialist colleagues.

## Coordinated Care Models for Secondary Fracture Prevention: Focus on FLS

Of ~ 2 million US individuals per year who sustain an osteoporotic fracture, < 20% receive follow-up care for osteoporosis [61]. A potential solution is coordinated osteoporosis care models [62], primarily FLS [63]. FLS aims to identify patients with recent osteoporotic fractures, assess their fracture risk, and implement interventions including fall risk mitigation, rehabilitation, pharmacotherapy, and other follow-up care [64]. FLS typically involves a care coordinator (e.g., physician assistant or nurse practitioner) who serves as a link between the orthopedic team, osteoporosis and falls services, the patient, and the primary care physician.

The AOA created the Own the Bone (OTB) program to empower orthopedic surgeons to implement and use FLS [55, 61]. Numerous other organizations also endorse FLS [4••, 6••, 63], which can deliver a variety of health care benefits [41, 61, 65, 66••, 67–72], including improvements in fracture ascertainment, DXA evaluation rates, secondary osteoporosis diagnosis, and recommendation and initiation of osteoporosis pharmacotherapy. Some studies show that patients managed via FLS experience lower secondary fracture rates [66••, 68, 70, 71] and lower mortality [68, 69, 72].

Patients managed via FLS are often receptive to osteoanabolic therapy [58••], and FLS-managed patients who were already taking antiresorptives show a willingness to switch to osteoanabolics after fracturing [73].

## Bone Health Assessment and Optimization in Orthopedic Surgery

FLS providers are ideally suited to foster perioperative bone health optimization (BHO), a growing trend for improving postoperative outcomes and reducing osteoporotic fractures and other complications in patients with poor bone health undergoing major bone surgeries, including joint replacement and spinal fusion. BHO helps identify and address suboptimal skeletal status in surgery patients [58••, 74, 75] via bone status assessment, identification and correction of metabolic deficits, and initiation of osteoporosis treatment when appropriate [60•]. Retrospective data from patients referred by surgeons for BHO before arthroplasty or thoracolumbar surgery indicated that 56% had prior fractures and 91% met the NOF criteria for osteoporosis pharmacotherapy; 75% of eligible subjects accepted therapy, two-thirds of whom were prescribed osteoanabolics [58••]. Preoperative and postoperative osteoporosis therapy each offer potential benefits [60•]. Retrospective data from individuals undergoing multi-level spinal fusion showed that preoperative osteoporosis pharmacotherapy was associated with lower odds of instrumentation complications, pathological fracture, and revision surgery [76].

BHO is recommended when considering major orthopedic surgery in patients aged ≥ 50 years old [60•] and for patients prior to elective orthopedic and spine surgery [77•]. For patients with osteoporosis who can tolerate postponement of spinal surgery, a consult with a bone health specialist may be warranted for the consideration of up to 6 months of preoperative osteoporosis therapy, with teriparatide preferable to bisphosphonates based on superior efficacy. For patients needing prompt surgery, consultation with a bone health specialist for postoperative osteoporosis therapy is advised [78]. We note that osteoanabolic therapy is only indicated to reduce fracture risk, which warrants at least 12 months of treatment (Fig. 2).

Various modalities and clinical criteria used in BHO to determine the need for and nature of interventions include DXA [39•, 40•, 74, 75, 77•, 79], DXA-based Trabecular Bone Score (TBS) [74], FRAX [74, 79, 80], and fracture history [60•, 80]. Liu et al. advises that hip or vertebral fractures indicate osteoporosis regardless of BMD [80]. Anderson et al. recommends lateral spine DXA or radiography to identify occult vertebral fractures [60•]. Quantitative computed tomography (QCT) can identify low spine BMD [81], and standard (phantomless) CT can identify low bone mass based on low Hounsfield units (HUs) [39•, 74, 77•, 82–84]. “Opportunistic” CT, whereby HUs are measured in CT scans performed for other clinical indications, is a growing practice in osteoporosis management that can identify low bone mass without additional radiation exposure [85]. This information can be easily and quickly obtained by the clinician, and an HU value below certain thresholds (e.g., < 90 or 100 HUs) can suggest osteoporosis, often leading to follow-up DXA for confirmation. CT can also be used to identify OVCFs, and recent data show that opportunistic spine CT identified ≥ 1 vertebral fracture in ~ 25% of all individuals aged ≥ 60 years [84]. A CT-based modality that is FDA-cleared for osteoporosis diagnosis is biomechanical CT (VirtuOst BCT; ON Diagnostics, Berkeley, CA) which uses finite element analysis to identify fragile bone strength, including in patients undergoing spinal fusion [86]. Starting with an opportunistic CT scan that captures the lower spine (without intravenous contrast) or hip (with or without contrast), physicians throughout the USA can order the BCT test via a centralized Medicare-enrolled diagnostic facility (https://ondiagnostics.com/order-virtuost/how-to-order-virtuost-tests/).

## Osteoporosis, Fracture Risk, and Bone Complications in Patients Undergoing Ortho/spine Surgery

### Spinal Fusion

Over one-third of individuals undergoing surgery for lumbar degenerative disease are ≥ 65 years old [87], and spinal fusion is the fourth-commonest surgery in the USA among individuals aged 65–84 years [88]. The risk of vertebral fractures and other bone complications after spinal fusion increases with age, osteoporosis, and low spine BMD [89–91]. These complications manifest earlier in patients with low BMD, often within the first postoperative year [91], and are more common in postmenopausal women [92]. In one recent study, only 14% of patients with osteoporosis by DXA who were undergoing ≥ 3-level spinal fusion received preoperative osteoporosis therapy; yet, the treated patients experienced fewer postoperative vertebral fractures, instrumentation complications, and revision surgeries [76]. In another study, ~ 40% of individuals aged ≥ 50 years undergoing lumbar fusion had osteoporosis based on DXA or CT, with an osteoporosis prevalence rate of nearly 80% in women aged ≥ 70 years; notably, spine CT identified potential osteoporosis (i.e., low CT-HUs) in > 25% of patients with non-osteoporotic DXA BMD *T*-scores [82]. Low preoperative CT-HUs predict osteoporosis-related complications after spinal fusion [93, 94], sometimes better than spine DXA [94], perhaps because CT can avoid regions with degenerative changes or vascular calcifications that can spuriously increase DXA BMD. CT-HUs can also reveal postoperative bone loss after lumbar fusion [83].

### Knee and Hip Arthroplasty

Patients undergoing TKA or THA are often older individuals with osteoporosis [95, 96] who have higher risks of suboptimal prosthesis fixation, prosthesis loosening, periprosthetic fractures, and osteoporotic fractures [80, 95–98]. Yet, osteoporosis evaluation and treatment before TKA/THA is rare [60•], partly due to erroneous perceptions that osteoarthritis protects against osteoporosis. In one study, > 50% of older TKA/THA candidates had osteoporosis by DXA [97]. Other data show that ~ 25% of TKA/THA patients had osteoporotic BMD *T*-scores preoperatively, only 1 in 4 of whom received perioperative osteoporosis treatment [95]. Among female TKA candidates with osteoporosis by preoperative DXA, < 14% received preoperative osteoporosis therapy [97]. While osteoporotic BMD is fairly common in older arthroplasty candidates, prior osteoporotic fractures can be even more common [96], and preoperative fracture history is associated with greater odds of periprosthetic fracture, prosthesis dislocation and instability, and secondary osteoporotic fractures after THA or TKA [54, 56•].

Many arthroplasty patients also experience rapid regional bone loss postoperatively. Meta-analyses of 14 TKA studies showed that ipsilateral distal femur BMD decreased postoperatively by 9.3% at 3 months and by 15.4% at 24 months, and that screening for low BMD and implementing preoperative BHO may mitigate the effects of postoperative bone loss [98].

## Effects of Osteoanabolics on Fracture Healing, Arthroplasty, Spinal Fusion, and Percutaneous Vertebral Augmentation

Osteoporosis increases the risk of bone-related complications in surgery patients, and pharmacotherapies that increase osteogenesis, decrease bone resorption, or both may contribute to better outcomes. Little level 1 evidence supports that notion, but few if any rigorous well-powered trials of sufficient duration have been conducted to exclude significant benefits. For some orthopedic patients with VHFxR, even “slight improvements” in bone healing via perioperative osteoanabolic therapy may be welcomed [99]. We emphasize that osteoporosis pharmacotherapy for orthopedic patients should be part of a long-term treatment plan to optimize bone health, which may facilitate surgical success in addition to the primary goal of reducing fracture risk.

### Fracture Healing

#### Teriparatide and Abaloparatide

PTHR agonists promote fracture healing in animals via early stimulation of chondrogenesis, continuous stimulation of osteogenesis, and accelerated callus remodeling [100]. Such effects may contribute to fracture healing benefits observed in some teriparatide clinical trials [101–103]. Recent meta-analyses on the effects of PTHR agonists on the healing of various fracture types indicate improvements in functional outcomes and pain but not in fracture healing rate or adverse events [104].

A randomized trial showed that teriparatide-treated subjects with acute OVCF had better early radiographic healing and disability scores than ALN-treated controls [105]. A non-randomized study of patients with acute unstable OVCF showed greater bony union by dynamic radiography and less vertebral collapse with teriparatide versus no-teriparatide controls [106]. A non-randomized retrospective study of postmenopausal women with OVCF showed less pain and disability and better physical performance with teriparatide versus calcium and vitamin D therapy [107]. A retrospective study of patients with OVCF showed better pain relief and fracture healing and less vertebral collapse with teriparatide (but not with bisphosphonates) compared with no pharmacotherapy [108].

A non-randomized study of patients with osteoporosis undergoing surgery for intertrochanteric hip fracture showed improved pain, function, and radiographic healing with 2 months of postoperative teriparatide versus no teriparatide [99]. Meta-analysis of teriparatide effects on hip fracture healing showed reduced time to union, but no effect on union rate at month 3 or month 6 or on complications, re-operation rate, or hip function [109]. A placebo-controlled randomized teriparatide trial for the healing of pelvic insufficiency fracture (PIF) was prematurely suspended, but data from 33 patients showed that both groups had similar improvements in radiographic healing and pain, and the teriparatide group had greater improvements in physical performance measures [110]. A meta-analysis of 2 randomized controlled trials and 6 case series of patients with PIF concluded that teriparatide had a positive effect of bone healing and functional outcomes [111]. The patient demographics and fracture types in these studies are suggestive of clinical osteoporosis. Teriparatide has also been evaluated as a treatment for nonunions and delayed unions, but most of those studies include many patients for whom such treatment would be off-label for lack of an osteoporosis diagnosis.

There are few reports to date on fracture healing with abaloparatide. Abaloparatide improves fracture healing parameters in rodent long bone fracture studies, including increases in callus osteogenesis and strength and improved bridging that correlates with the extent of early callus cartilage [112, 113]. Modest osteoclast stimulation and remodeling activation with abaloparatide may also favor primary (osteonal) healing and callus remodeling. A prospective, randomized, double-blind phase 2 trial is underway in ≥ 50-year-old women and men to study the effects of abaloparatide versus placebo on the healing of acute PIFs (ClinicalTrials.gov: NCT04249232).

#### Sclerostin Inhibition (Romosozumab)

The effects of romosozumab on hip fracture healing was studied in a prospective phase 2 trial of 332 patients (66% female, mean age 79 years) with recent intertrochanteric or femoral neck fractures treated with open reduction and internal fixation [114]. Patients received various doses of romosozumab or placebo on day 1 and at week 2, 6, and 12 postoperatively. There were no differences between any romosozumab dose group versus placebo for the timed up-and-go test (primary outcome), radiographic healing, or RUSH (Radiographic Union Score for Hip) scores. Cardiovascular and fatal adverse events were numerically greater in the group receiving romosozumab at the EVENITY® dose (210 mg) versus placebo. Lack of romosozumab efficacy on fracture healing could potentially reflect over-riding effects of internal fixation that fostered robust healing independent of romosozumab treatment. But, anti-sclerostin antibodies (Scl-Ab) may also have biological limitations for fracture healing, including an apparent lack of early pro-chondrogenic effects that may limit their ability to promote cortical bridging [100], and an antiresorptive effect that could limit osteonal healing.

### Spinal Fusion

Spinal fusion can provide meaningful pain relief, but these surgeries also carry risks, especially in patients with osteoporosis, including pseudoarthrosis, instrumentation failure, adjacent segment disease, and postoperative vertebral fractures [115]. Antiresorptives can reduce vertebral fracture risk after spinal fusion, but antiresorptives do not promote osteogenesis or chondrogenesis, and antiresorptives are less effective than teriparatide for reducing postoperative complications and promoting arthrodesis [116, 117•, 118], an endochondral process [119].

Spinal fusion studies show that teriparatide increases histomorphometric parameters of bone formation within 3 months [120] and spine BMD within 6 months [121]. In a randomized controlled trial in patients with osteoporosis undergoing instrumented posterior lumbar interbody fusion (PLIF), postoperative teriparatide users had greater odds of fusion at 6 months and lower odds of developing spondylolisthesis versus non-users [122]. Retrospective data from patients undergoing instrumented transforaminal lumbar interbody fusion (TLIF) showed that 3 months of preoperative teriparatide and continued postoperative teriparatide was associated with preservation of adjacent level bone mass and higher fusion scores versus no teriparatide; both groups had similar clinical outcomes and complications [83].

A randomized double-blind study in patients (mean age ~ 70–71 years) undergoing non-instrumented posterolateral fusion (PLF) showed that 90 days of postoperative teriparatide did not enhance arthrodesis or fusion mass versus placebo [123]. Patients were not selected based on low BMD, but this study is mentioned as a rare source of level 1 evidence on the effects of osteoporosis pharmacotherapy on arthrodesis. Lack of teriparatide efficacy could relate to the lack of instrumentation, reliance on suboptimal bone graft (from cortex-rich laminectomy bone rather than cancellous-rich iliac crest bone), the inclusion of patients without poor bone health, or the relatively brief duration of therapy. A non-randomized prospective study of ≥ 50-year-old patients with low bone mass undergoing spinal fusion for various conditions showed that patients who received ≥ 3 months of preoperative (and optional postoperative) teriparatide had fewer osteoporosis-related complications (screw loosening, cage-adjacent radiolucency, rod fracture, or new vertebral fracture) and better disability scores versus those who declined teriparatide [120]. In a retrospective study of patients with osteoporosis undergoing lumbar fusion, pre- and postoperative teriparatide users had lower 2-year odds of osteoporosis-related complications (adjacent segment disease, pseudoarthrosis, readmissions, and reoperation) versus non-users [124].

A randomized trial in women with PMO undergoing instrumented PLIF showed higher 6-month fusion rate and 12-month femoral neck BMD gains with teriparatide versus ZOL; both groups showed similar fusion rates at months 12–24 and similar clinical outcomes at month 24 [125]. A retrospective study of patients undergoing instrumented ≥ 3-level fusion surgery for OVCFs showed a lower incidence of vertebral fractures among pre- and postoperative teriparatide users versus bisphosphonate users [126]. Retrospective analyses of patients with osteoporosis undergoing TLIF showed higher fusion rate and greater spine BMD at 1 year in those receiving postoperative teriparatide for ≥ 6 months versus those who received ≥ 1 annual infusion of ZOL; pain and disability scores were similar in both groups [127]. A retrospective study of patients undergoing instrumented PLF for OVCF showed fewer mechanical complications (i.e., new vertebral fractures, screw complications, rod fracture, pseudoarthrosis) among those using postoperative teriparatide versus bisphosphonates [128].

No published clinical data on abaloparatide or romosozumab in spinal fusion were identified. Abaloparatide increased bone formation, fusion mass density, and arthrodesis in rat and rabbit PLF models [129, 130], and an ongoing randomized trial in postmenopausal women is studying the effects of 6 months of preoperative abaloparatide versus placebo on the success of single- or multi-level PLF surgery (ClinicalTrials.gov: NCT03841058).

One rat PLF study showed that Scl-Ab increased fusion mass but not arthrodesis rate [131], while another showed that Scl-Ab increased arthrodesis and osteophytes [132].

### Vertebral Augmentation

Vertebroplasty (VP) and balloon kyphoplasty (BKP) are cement augmentation approaches for treating painful VCFs. These percutaneous procedures reduce pain versus non-surgical care, and BKP can also restore vertebral height. Some clinical trials showed no clinically significant benefits of VP/BKP versus sham procedures, though recent data support the use of VP/BKP in selected patients [133]. The risk of adjacent-level fractures increased after VP/BKP in some studies [134] but not in others [59]. An ASBMR Task Force Report on vertebral augmentation concluded that BKP provides some clinical benefit over non-surgical management, with osteoporosis pharmacotherapy reducing subsequent vertebral fractures by 40–70% [135•].

Several recent studies evaluated teriparatide in patients undergoing VP/BKP; no published VP/BKP studies with abaloparatide or romosozumab were identified. A prospective study of postmenopausal women with OVCFs treated with VP plus ALN versus conservative teriparatide alone showed similar clinical improvements in both groups after 1–3 months, with the VP plus ALN group showing better pain reduction at week 1 and greater vertebral height restoration at month 3 [136•]. A retrospective study of postmenopausal women with OVCFs showed similar function outcomes after ≥ 6 months of conservative teriparatide therapy versus spinal fusion, and patients treated with spinal fusion or teriparatide plus VP showed better vertebral morphology restoration and short-term functional outcomes versus conservative teriparatide [137].

Other retrospective studies report the following: teriparatide after BKP is associated with fewer VCFs, increased spine BMD, better health-related quality of life scores, and less back pain versus patients receiving calcium and vitamin D [138]; teriparatide after BKP is associated with fewer VCFs versus control subjects who mostly received ALN [139]; and teriparatide after VP is associated with greater vertebral body height and reduced refracture rate versus VP without teriparatide [140].

### Hip and Knee Arthroplasty

Local bone resorption can lead to subsidence, loosening, and failure of prosthetic implants, and recent data expand evidence that the use of potent antiresorptives after TKA or THA reduces periprosthetic osteolysis and related sequelae in patients with osteoporosis [141–144]. Osteoanabolics have greater potential to rapidly improve bone stock preoperatively, which improves primary implant stability in animals [145]. Postoperative use of osteoanabolics may also promote implant osseointegration, as shown by increased bone-implant contact and pull-out strength in animals treated with teriparatide [146], abaloparatide [113], or Scl-Ab [145, 147]. The ability of Scl-Ab to stimulate bone formation and inhibit resorption may have particular value for promoting and maintaining implant osseointegration [147].

Few high-quality studies show improved clinical outcomes with osteoanabolics after arthroplasty, but favorable effects of osteoanabolics on surrogate endpoints are evident, including peri-implant BMD and implant migration. A prospective study in women with PMO undergoing cementless TKA showed that 12 months of postoperative teriparatide increased periprosthetic BMD versus no-teriparatide controls [148]. Retrospective database analyses of patients with recent fragility fractures prior to TKA showed a lower risk of secondary osteoporotic fractures among perioperative TPTD users (but not bisphosphonate users) versus untreated controls [56•].

An open-label study is currently ongoing to evaluate the effects of 18 months of abaloparatide starting 3 months preoperatively in women and men with osteoporosis undergoing TKA, with clinical endpoints including regional BMD changes and knee function scores (ClinicalTrials.gov: NCT04167163).

## Future Directions and Summary

Better identification of patients with VHFxR may be aided by broader use of opportunistic CT (including at non-vertebral sites) and by further clinical validation of CT-HU thresholds for predicting fracture risk. Establishment of order sets that automate the identification of various VHFxR criteria is another worthy goal.

Future BHO advancements may include the establishment of “bone health thresholds” that indicate the need for surgical delay for BHO. More high-quality data would help define the impacts of osteoporosis on functional outcomes after arthroplasty [80]. Better standardization of periprosthetic DXA/CT regions would help in evaluating the efficacy of osteoporosis therapies on implant fixation [98]. More data are needed to optimize the perioperative use of osteoporosis therapies, including the minimum effective duration of preoperative therapy based on yet-to-be-established success criteria. More placebo-controlled trials of osteoanabolics in patients undergoing spinal fusion would fill an important evidence gap [118]. Implementation of opportunistic CT-based bone quality assessments into surgical navigation algorithms could allow identification of poor bone quality and real-time adjustment of surgical plan, while also guiding postoperative management.

In summary, osteoanabolic pharmacotherapies play important roles in secondary fracture prevention and may have additional potential to promote bone regeneration and healing in patients with recent osteoporotic fractures and in patients with osteoporosis undergoing major bone surgeries, including arthroplasty and spinal fusion. Osteoanabolic agents are much more expensive than antiresorptives, but their use by such patients may be appropriate based on pharmacoeconomic and humanistic considerations. Surgeons are encouraged to implement and utilize FLS to efficiently administer follow-up care to post-fracture patients with osteoporosis. Surgeons should also consider BHO for surgery candidates suspected of having suboptimal bone health or found to have poor bone status intraoperatively. Table 1 provides a snapshot of patient features to help surgeons recognize patients that may benefit from a bone health evaluation conducted by themselves, FLS, or bone health specialist colleagues.

## Footnotes

1 Abbreviations for various societies and organizations are defined in Table 1 footnotes.
